# Novel rearrangements between different chromosomes with direct impact on the diagnosis of 5p- syndrome

**DOI:** 10.1016/j.clinsp.2022.100045

**Published:** 2022-05-28

**Authors:** Samar Nasser Chehimi, Vanessa Tavares Almeida, Amom Mendes Nascimento, Évelin Aline Zanardo, Yanca Gasparini de Oliveira, Gleyson Francisco da Silva Carvalho, Beatriz Martins Wolff, Marilia Moreira Montenegro, Nilson Antônio de Assunção, Chong Ae Kim, Leslie Domenici Kulikowski

**Affiliations:** a Laboratório de Citogenômica, Departmento de Patologia, Faculdade de Medicina, Universidade de São Paulo (FMUSP), São Paulo, SP, Brazil; b Unidade de Genética, Departamento de Pediatria, Instituto da Criança, Hospital das Clínicas, Faculdade de Medicina, Universidade de São Paulo (HCFMUSP), São Paulo, SP, Brazil; c Escola Paulista de Medicina, Universidade Federal de São Paulo, São Paulo, SP, Brazil; d Departamento de Química, Instituto de Ciências Ambientais, Químicas e Farmacêuticas, Universidade Federal de São Paulo, Diadema, SP, Brazil

**Keywords:** Genomic rearrangements, Microarray, 5p deletion, Copy number variation

## Abstract

**Objectives:**

Copy Number Variations (CNVs) in the human genome account for common populational variations but can also be responsible for genetic syndromes depending on the affected region. Although a deletion in 5p is responsible for a syndrome with highly recognizable phenotypical features, other chromosomal abnormalities might overlap phenotypes, especially considering that most studies in 5p use traditional cytogenetic techniques and not molecular techniques.

**Methods:**

The authors have investigated 29 patients with clinical suspicion of 5p- syndrome using Chromosomal Microarray (CMA), and have gathered information on previous tests, clinical signs, symptoms, and development of the patients.

**Results:**

The results showed 23 pure terminal deletions, one interstitial deletion, one deletion followed by a 3 Mb duplication in 5p, three cases of 5p deletion concomitant to duplications larger than 20 Mb in chromosomes 2, 9, and 18, and one 5p deletion with a chromosome Y deletion. CMA showed relevant CNVs not typically associated with 5p- that may have contributed to the final phenotype in these patients.

**Conclusions:**

The authors have identified three novel rearrangements between chromosomes 5 and 2 (Patient 27), 5 and 18 (Patient 11), and 5 and Y (Patient 22), with breakpoints and overlapped phenotypes that were not previously described. The authors also highlight the need for further molecular investigation using CMA, in different chromosomes beyond chromosome 5 (since those cases did not show only the typical deletion expected for the 5p- syndrome) to explain discordant chromosomal features and overlapped phenotypes to unravel the cause of the syndrome in atypical cases.

## Highlights

• The authors The authors have described three novel rearrangements between chromosomes 5 and 2, 5 and 18, and 5 and Y with chromosomal breakpoints and overlapped phenotypes that were not previously described.
• One of the main atypical features for 5p- syndrome that the authors report was the presence of seizures that was found in the three patients with rearrangements between different chromosomes and in a patient with a deletion followed by duplication in 5p.
• The authors suggest physicians conduct further molecular investigation in the presence of atypical clinical features for patients with 5p- syndrome suspicion.

## Background

It is a well-known fact that deletions of variable sizes in the short arm of chromosome 5 cause a syndrome known as Cri du Chat syndrome, called after the high-pitched cat-like cry that patients present since birth, or 5p- syndrome. The incidence of this syndrome in the general population is 1/15,000 to 1/50,000 live births.^1^^,^^2^ Of the total of 5p- cases, 80‒90% are *de novo* cases, about 10‒15% are inherited from balanced translocations in one of the patient's parents (usually paternally inherited), and the rest of the cases arise from ring chromosomes, inversions and other structural rearrangements.^1^^,^^3^

The consequences of this syndrome involve the presence of Intellectual Disability (ID), difficulty breastfeeding (poor suction), difficulty gaining weight during the first years, failure to thrive and communicate verbally, short stature, and also some facial features such as microcephaly, ocular hypertelorism, short philtrum and microretrognathia.^1^^,^^3^ Phenotypical features, in addition to the ones previously described, can be a strong indication of a possible rearrangement between chromosome 5 and another chromosome(s) that leads to phenotype overlap and requires a deeper molecular investigation.

The main cytogenetic technique used in Brazil to investigate suspicion of 5p- syndrome is the G-banded karyotype. Although most of the cases involve deletions larger than 10 Mb and can be seen using the G-banded karyotype, several cases of translocations with other chromosomes remain unsolved, either because of the G-banding pattern or the size of the deletions. In these cases, other molecular techniques are needed. Fluorescence In Situ Hybridization (FISH) and Multiplex Ligation-dependent Probe Amplification (MLPA) is straightforward and precise tools for molecular cytogenetics. However, both require a previous suspicion of the chromosomes that can be involved in the rearrangement for the selection of the target probes or kits, respectively.^4^ Chromosomal Microarray (CMA) allows the detection of Copy-Number Variations (CNV) across the entire genome without focusing on specific chromosomal targets, allowing the visualization of concomitant deletions or duplications to the 5p deletion causative of the syndrome with high resolution of thousands of genomic targets simultaneously.^4^^,^^5^

In this study, the authors analyzed CMA results for 29 patients with confirmed 5p- deletion using cytogenetic molecular testing, and highlighted one case of a deletion followed by duplication in 5p with an unusual phenotype and three atypical cases of complex rearrangements unsolved by the karyotype that required CMA for the complete molecular diagnosis and that were not previously described.

## Methods

A cohort of 29 patients with a clinical diagnosis of 5p- syndrome (20 females and 10 males) were selected for this study. All enrolled patients were invited for this study during clinical evaluation by geneticists at the Unit of Clinical Genetics ‒ *Instituto da Criança, Hospital das Clínicas ‒ Universidade de São Paulo* (ICr‐HCFMUSP) or in the annual national meetings of families of 5p- carriers held in the city of São Paulo between 2015‒2019. The inclusion criteria were the presence of a high-pitched cry since birth or the detection of the 5p deletion using G-banded karyotype, MLPA, FISH, or chromosomal microarray.

To perform CMA, the authors started with an initial input of 20 ng/μL concentration of DNA. Genomic DNA was isolated from peripheral blood lymphocytes using a commercially available DNA isolation kit (QIAamp DNA Blood Mini Kit®, Qiagen) following the manufacturer's instructions. The quality and quantity of the DNA samples were determined using Qubit Fluorometer (Invitrogen), and the integrity of the DNA was ascertained via agarose gel electrophoresis analysis.

CMA was performed for all samples. Twenty-five samples were performed using the Infinium CytoSNP‐850K BeadChip, and four samples were analyzed through other platforms.

Illumina's recommended protocol was followed, and the raw data were analyzed using BlueFuse™ Multi v4.4 (Illumina, Inc.). The genomic positions are given as mapped to the GRCh37/hg19 genome build.

All the CNVs detected were analyzed according to the most recent American College of Medical Genetics guidelines^5^ and classified as benign, likely benign, VUS (variants of uncertain clinical significance), likely pathogenic, or pathogenic according to the following databanks: the Database of Genomic Variants (DGV, https://projects.tcag.ca/variation/), the Database of Chromosomal Imbalance and Phenotype in Humans Using Ensembl Resources (DECIPHER, http://decipher.sanger.ac.uk/), The Clinical Genome Resource (ClinGen) the UCSC Genome Bioinformatics database (http://genome.ucsc.edu), National Center for Biotechnology Information (NCBI, http://www.ncbi.nlm.nih.gov/) and Pubmed for publications. The genomic positions are reported according to their mapping on the GRCh37/hg19 genome build.

The following criteria were used to filter the CNVs in BlueFuse™ Multi: no predefined minimum size for a CNV, the need for at least ten consecutive deleted or duplicated probes to assume a CNV as real, ratio cutoff values considered valid when log_2_ ratio is at least −0.41 for deletions and +0.32 for duplications and a minimum size of 3 Mb and 500 consecutive probed modified to consider it a Region of Homozygosity (ROH).

## Results

Previously, the authors have reported the results of 14 patients with different deletions in 5p that enabled the detection of a new genomic region that might be associated with the high-pitched cry and microcephaly.^6^ Now, with a bigger cohort (a total of 29 patients, including the 14 that were studied before), the authors have identified new results that bring to light more details about the chromosomal features that can be found in patients with 5p- syndrome.

After CMA was performed, the authors detected pure terminal deletions in 23 patients, one interstitial deletion in 5p, one deletion followed by a 3 Mb duplication in 5p (Patient 5), three cases of deletion concomitant to a large duplication (>20 Mb) between chromosomes 5 and 9 (Patient 9), 5 and 2 (Patient 27), 5 and 18 (Patient 11), and one case of 5p deletion with a chromosome Y deletion (Patient 22).

### Patient 5

**Fig. 1:**
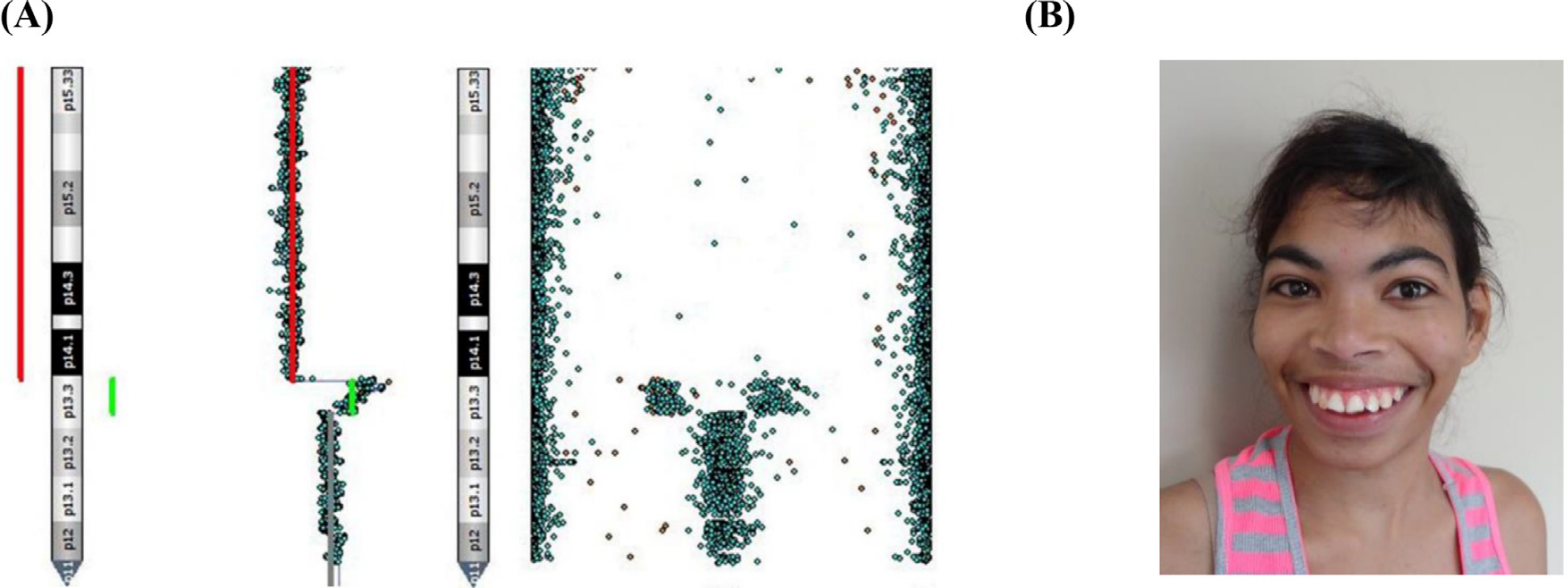
Representation of the deletion followed by a duplication in 5p, found in Patient 5, obtained with the software BlueFuse for Illumina's chromosomal microarray analysis (A) and a picture of the patient's face, at the age of 22-years and 5-months (B).

Patient 5 presented, in addition to the 5p deletion, a duplication in the region adjacent to the breakpoint of the deletion [her CMA result was arr[GRCh37] 5p15.33p13.3(25328_29305055)x1, 5p13.3(29320222_32402232)x3] (Fig. 1A). The clinical features of this patient were ocular hypertelorism, epicanthal folds, dysplastic ears, short philtrum, high palate, microretrognathia, hypotonia, and seizures (Fig. 1B).

### Patient 11

**Fig. 2:**
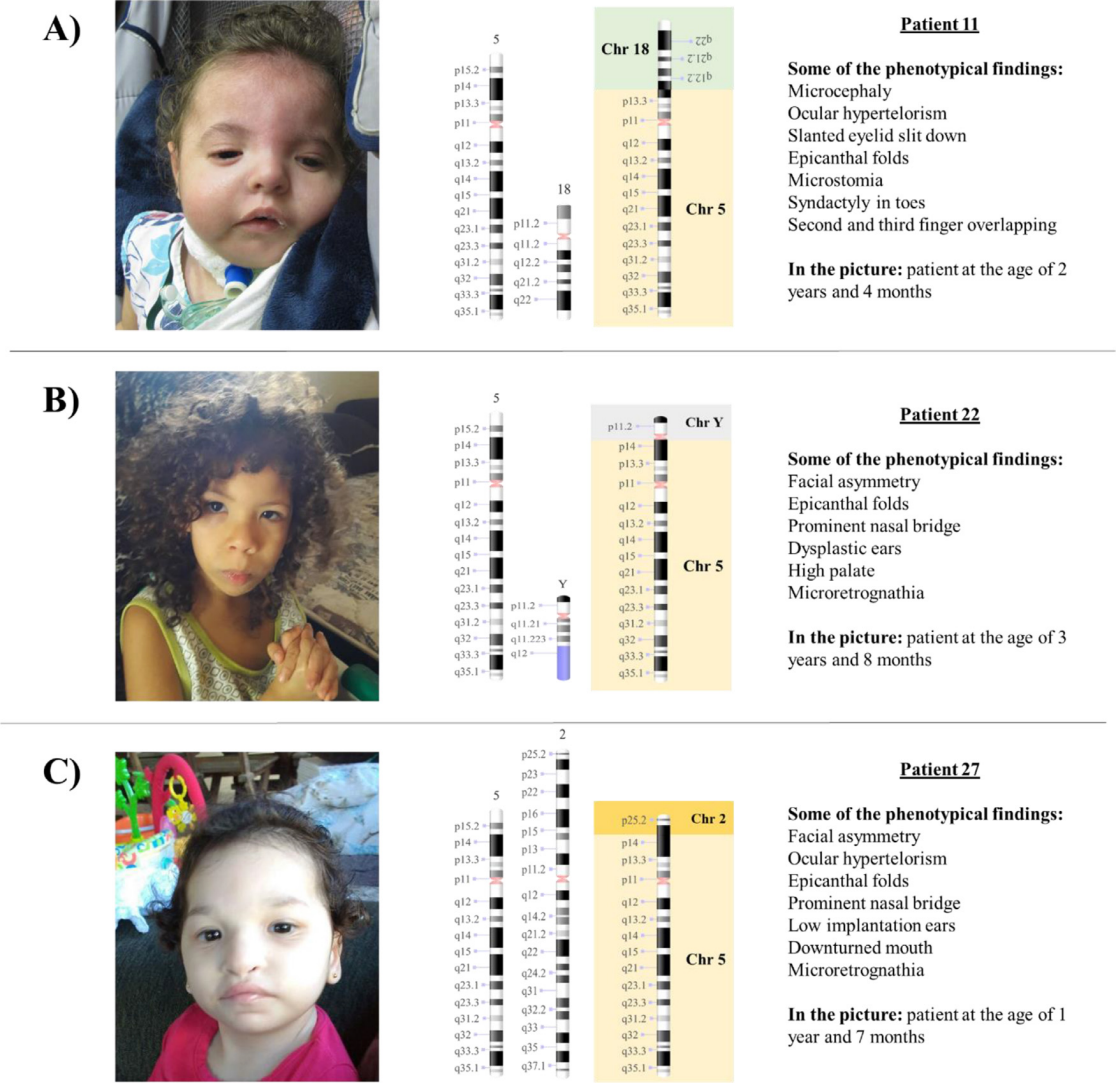
Pictures of patients' faces, followed (at the right side) by the chromosomal representation of the rearrangements observed with the phenotypical findings that were addressed individually in the discussion. (A) Patient 11 at the age of 2-years and 4-months, with a rearrangement between chromosomes 5 and 18, generating a phenotype compatible with 5p- syndrome and 18q duplication syndrome, (B) Patient 22 at the age of 3-years and 8-months, with a rearrangement between chromosomes 5 and Y, that led to an initial unexpected karyotype of 45,X, based on the normal male genitalia and clinical features that matched 5p- syndrome, and (C) Patient 27 at the age of 1-year and 7-months, with a rearrangement that generated a derivative chromosome 5, originated from 5p deletion and 2p duplication. All images for the chromosomal rearrangements were constructed using NCBI Genome Decoration Page.

It was identified that Patient 11 inherited a derivative chromosome from a maternal complex translocation rearrangement involving chromosomes 5, 10 and 18 [the mother's karyotype was 46,XX,t(18;5;10)(18qter->18q11.1::5p11.1;inv(5)(5pter->p(11.1)::10q11.1;10qter->10q11.1::18q11.1)]. The combination of data available about previous results and the results after CMA provided some insights into the architecture of the derived chromosome, as shown in Fig. 2A.

Patient 11 is the daughter of healthy non-consanguineous parents. During pregnancy, a 20-week ultrasound detected intrauterine growth restriction, and at 24-weeks, pericardial effusion was detected. At 28-weeks, a fetal echocardiogram suggested the presence of tetralogy of Fallot. Then it was detected muscular ventricular septal defects and interatrial communication (oval fossa defect). The patient was born at 36-weeks with neonatal jaundice, hypotonia at birth, difficulty breastfeeding, low oxygen saturation, and brief shaking episodes in upper and lower members (an epileptiform activity that required medication). Among her recent previous health conditions, she had recurrent respiratory infections and bronchoscopy, and had to use supplemental oxygen (0.1 L/min).

This patient presented severe developmental delay (cannot verbalize, speak, or understand signs and gestures), microcephaly, ocular hypertelorism, slanted eyelid slit down, epicanthal folds, short filtrum, microretrognathia, microstomia, gastroesophageal reflux, mild clinodactyly, syndactyly in toes, second and third finger overlapping.

### Patient 22

At birth, Patient 22 presented high-pitched cry, difficult breastfeeding, and ocular hypertelorism. During his first year, several clinical tests were conducted: brain MRI showed hypoplasia of the corpus callosum with mild prominence of the lateral ventricles. Other types of testing were conducted (Doppler echocardiography, electroencephalogram, visual evoked potential, abdominal ultrasound, transfontanellar ultrasound, and brain tomography), showing no alterations. During childhood, two episodes of pneumonia, several episodes of bronchitis and throat infections were described by the mother.

His clinical features included facial asymmetry, epicanthal folds, prominent nasal bridge, dysplastic ears, high palate, microretrognathia, and gastroesophageal reflux. All features mentioned are expected for 5p- syndrome.

### Patient 27

Patient 27 presented low weight at birth and a high-pitched cry. Several episodes of pneumonia and recurrent respiratory infections during early childhood were mentioned by the mother. The patient cannot communicate verbally, but she understood commands and could communicate using some specific signs. She presented facial asymmetry, ocular hypertelorism, epicanthal folds, prominent nasal bridge, low implantation ears, downturned mouth, and microretrognathia. She presented congenital heart abnormalities such as bicuspid aortic valve, interatrial communication, and ventricular septal defect.

## Discussion

As previously reported, the authors have detected some interesting features about a phenotype-genotype association and inferred a new region as responsible for the cat-like cry and evidence of microcephaly.^6^

A deeper investigation, including more patients with 5p- syndrome phenotype, has revealed rearrangements between chromosome 5 and other autosomes (chromosomes 2 and 18) and Y chromosome that was not previously reported, bringing light to the possibility of new genomic rearrangements involved in 5p- syndrome, usually investigated alone with no further inspection in other co-occurring CNVs.

The short arm of chromosome 5 contains approximately 120 protein-coding genes.^7^ Among the most studied genes in 5p, some of them present interesting characteristics for this study. *SEMA5A* and *CTNND2* can lead to ID and autistic spectrum behaviors when both genes are deleted.^8^^,^^9^ Likewise, the genes of the cadherin family (*CDH18, CDH12, CDH10, CDH9, CDH6*), deleted in varying proportions in patients with 5p, also suggest the high susceptibility to the manifestation of the autistic spectrum.^10^

The authors have identified that all patients presented ID, but autism could not be assessed since the patients were not evaluated by psychologists and specialists in autism. But yet, some features that the authors have identified as the presence of irritability in 70.4% (19/27) of the patients, self-injury in 67.9% (19/28), and aggressive behaviors in 57.1% (16/28) might be an indication of autism traits.

Mutations in *TRIO* are associated with ID with microcephaly in an autosomal dominant pattern. This gene is highly expressed in the developing brain. Intragenic mutations, with consequent loss of function, have been associated with moderate intellectual disability combined with characteristics of the autistic spectrum, hyperactivity, and/or aggressive behavior. In addition, Nguyen et al.^7^ have shown cases of five individuals from four families with *TRIO* mutations that presented recurrent infections probably due to decreased expression of this gene in the early stages of neuronal development and have also shown that the knockdown of *TRIO* in rats impact on the development of neurites, filaments originating from neuronal cells, and synapse formation.^11^^,^^12^ In this study, 27 patients had a deletion in this gene, but yet there's no available data in the literature to understand how CNVs impact the expression of *TRIO*, only a few reports about specific variants.

### Patient 5

Concomitant duplications and deletions on the same chromosome, as seen in Patient 5, have already been identified on other autosomal chromosomes and may be associated with overlapping features.^13^ 5p trisomy is a rare syndrome, varying from the 5p15 portion to the short arm of the entire chromosome, with few reports available. The critical region of this duplication syndrome comprises the 5p10–5p13.1 bands, being mainly responsible for speech delay, intellectual disability, microcephaly, ear malformations, cardiac and brain malformations, alterations in limbs and ligaments, hypotonia, seizures, among others. Several available reports often point to rearrangements involving deletions followed by duplications on chromosome 5.^14^, ^15^, ^16^

Patient 5 was one of the four patients in this study who had seizures, which might be associated with 5p duplication since seizures are not often described for 5p- syndrome. Kluger et al.^17^ reported the case of two patients who had generalized epilepsy, and its chromosomal analysis showed duplications in different regions in 5p, with the 5p13.1 region overlapped in both situations. The authors speculated about the pathogenicity of the overlapping region, pointing out that the *FYB* gene could be involved in epileptogenesis, despite the fact that it was inherited from a healthy father and without this feature. Therefore, although Patient 5 in this study does not have the 5p13.1 region involved in duplication, restricting duplication to the 5p13.3 region alone, the authors cannot rule out the responsibility of this region in the occurrences of epilepsy since data on duplication of 5p and its association with epilepsy are still lacking.

### Alterations in 5p concomitant to other regions

The authors have detected four cases of patients (9, 11, 22, and 27) that showed 5p- concomitant to other large CNVs, ranging from 20.6 Mb (Patient 9) to 48.2 Mb (Patient 11), from different chromosomal origins (Table 1). The pictures of the patients and rearrangements are shown in Fig. 2.

**Table 1:** Concomitant alterations detected with 5p in Patients 9, 11, 22, and 27. All patients used G-banded karyotype and CMA for molecular investigation and Patients 9 and 22 also included MLPA and FISH for a satisfactory result. None of the four patients showed a typical 5p deletion expected to be seen on a karyotype for the suspicion of 5p- syndrome. After CMA, the deletion of 5p was observed along with other CNVs.

Patient	Previous results (KT, MLPA and/or FISH)	Results after CMA
9	KT: 46,XY	arr[GRCh37]5p15.33p14.1(25328_25351609)x1; 9p24.3p21.3(46587_20642438)x3
	MLPA P036-E1: rsa 5p(PDCD6)x1; rsa 9p(DMRT1)x3	
	FISH: 46,XY.ish der(5)t(5;9)(p15;p24)(189N21-,43N6+)	
11	KT: 46,XX,add(5)(p15.3)	arr[GRCh37] 5p15.33p14.2(113576_24272820) × 1; 18q12.1q23(29782990_78013728) × 3
22	KT: 45,X	arr[GRCh37] 5p15.33p15.1(22149_17812007)x1; Yq11.21q12(14852740_59335913)x0
	MLPA P064/P036/P070: rsa 5p(TERT, CLPTM1L, IRX4, CTNND2, PDCD6, CCDC127)x1; rsa Y-PAR2(VAMP7)x1	
	FISH: 45,X.ish X(DXZ1+),der(5)(SRY+)	
27	KT: 46,XX	arr[GRCh37] 2p25.3p24.1 (12770_23337574)x3; 5p15.33p15.1(113576_15713132)x1

Abbreviation: CMA, Chromosomal Microarray; KT, G-banded Karyotype; MLPA, Multiplex Ligation Probe Amplification; FISH, Fluorescence In Situ Hybridization.

An interesting fact is that those four patients presented seizures, which are not a typical manifestation of 5p- syndrome. Once the authors performed the CMA analysis, other CNVs candidates were detected as the causative origin of the seizures. Patient 9, with a 20.6 Mb duplication in 9p, was previously reported.^6^^,^^18^ Each one of the other three cases has not been described before in the literature and will be approached individually.

### Patient 11

This is a unique case regarding the breakpoints of a 24.2 Mb 5p deletion (5p15.33p14.2) and a 48.2 Mb 18q duplication (18q12.1q23) (Fig. 2A), that were not reported before and highlighted the need for a combination of techniques to have an accurate result.

Other cases of complex chromosome rearrangements maternally inherited have been reported before. Pan et al.^19^ have shown a patient with rearranged chromosomes 3 and 8, with duplication of 35.4 Mb in the 15q21.3-q26.2 region, that was inherited from the mother who had a complex rearrangement between chromosomes 3, 5, 8, 11 and 15. The present study's patient presented phenotypic overlap between 5p deletion and 18q duplication.

18 trisomy is a well-described syndrome, also known as Edwards syndrome. The main characteristics of the syndrome include facial and skeletal dysmorphisms with emphasis on the claw closed hand, with overlapping fingers that are generally underdeveloped. They also present feeding difficulties, congenital heart and kidney abnormalities.^20^ The phenotypic overlap of both syndromes, seen in the present case, resulted in a wheelchair-dependent patient, who is tracheostomized, does not communicate verbally or with gestures, and shows a delay in neuropsychomotor development. In addition, she also presented episodes of seizures. Several types of epilepsy have already been reported as a common feature of 18q duplication, with a prevalence of 65%.^21^

### Patient 22

Patient 22 is a 5-years old boy that presents a 17.8 Mb deletion in 5p (5p15.33p15.1) and 44.5 Mb deletion in Yq (Yq11.21q12). The remaining chromosomal fragment of the Y chromosome was translocated to the deleted region in 5p (and very likely to have generated a dicentric chromosome based on the breakpoints), indicating a karyotype of 45, X, that did not match the visible normal male genitalia nor the clinical features for 5p- syndrome of the patient. After karyotype, MLPA, FISH, and CMA were conducted, the rearrangement was uncovered (Fig. 2B).

This is a case of clinical relevance because few cases of men with translocations between chromosomes 5 and Y were published over the past 30 years, one of which is described in a mosaic [45, X, del(5)(p14)/45, X,t(Y;5)(q11;p14)].^22^^,^^23^

Generally, microdeletions on the Y chromosome are associated with male infertility and azoospermia.^24^ Previous studies involving translocations between chromosomes 5 and Y do not point to any specific changes associated with the Y deletion (including reports mentioning normal external male genitalia and no other abnormalities). Previous studies also pointed to phenotypic characteristics commonly associated with 5p-, such as hypertelorism and micrognathia, except for a report that cites a malformation of the left hand,^22^ but which is probably due to unchecked or not reported chromosomal abnormality other than 5p-. The authors have seen typical features of 5p- syndrome in the present study's patient but no investigation about infertility was conducted (the patient is too young) to confirm the clinical impact of the Y deletion and rearrangement in this case. Our case corroborates previous phenotypic findings with greater molecular detail and an update on the detection of translocations between chromosomes 5 and Y.

### Patient 27

The last case of rearranged chromosomes was Patient 27, a 5-years old girl that initially had a 46, XX G-banded karyotype result, and only by using CMA it was possible to observe that, in fact, she presented a 15.6 Mb 5p deletion (5p15.33p15.1) and a 23.3 Mb 2p duplication (2p25.3p24.1), rearranged in a way that the 2p duplication was positioned in the deleted segment of 5p and could not be distinguished earlier because the banding pattern on G-banded karyotype was similar to what is expected for the short arm of chromosome 5 (Fig. 2C)

There is not much literature available on the phenotypic effect of isolated duplication of 2p in patients, and it is usually seen with other chromosomal alterations. The phenotypes described in individuals with 2p duplication are associated with low weight, hypertelorism, low nasal bridge, ocular malformations, small jaw, low implantation ears, short neck, difficulty breathing, among others,^25^ and some features that were in accordance with what the authors found in Patient 27.

A case similar to ours regarding cytogenomic findings is the report of molecular characterization of two brothers with the trisomy of 2p24.3-pter and monosomy of 5p14.3-pter, inherited in a paternal manner, in which one of the brothers has spina bifida as a defect of the formation of the neural tube.^26^ Another study mentions a translocation between chromosomes 2 and 5 in a patient with obesity due to hyperphagia, however, involving a microdeletion in 2q23.1 and not in 2p.^27^ None of the two main characteristics presented above, spina bifida and obesity, is present in our patient, indicating how complex the interaction between this molecular rearrangement is.

## Conclusion

Previous studies carried out to identify deletions in 5p used classical cytogenetics techniques, such as karyotype and FISH, as breakthrough analysis devices. CMA stands out for a more precise delineation of breakpoints, revealing small CNVs not detected on G-banded karyotypes and allowing the inference of rearranged chromosomes inherited from balanced translocations or originated *de novo*, as the authors have shown in atypical cases. Here, the authors presented the cases of three patients with classical clinical findings for 5p- syndrome (Patient 22) or overlapped and atypical phenotype, as the presence of seizures (Patient 11 and 27), but without the molecular rearrangements expected for the syndrome. This emphasizes that this syndrome can manifest in other forms rather than the pure 5p deletion, and full molecular investigation in rare syndromes must be conducted by geneticists and physicians to clarify clinical features observed in patients with unexplained karyotypes or phenotypic overlap.

## Statement of ethics

The study was approved by the Institutional Review Board Ethics Committee for Analysis of Research Projects HCFMUSP/CAPPesq (CAAE: 62322416.7.0000.0068) and written consent was obtained from all the participants and/or their parents.

## Data availability

All data generated or analyzed during this study are included in this article and its supplementary material files. Further inquiries can be directed to the corresponding author.

## Funding

This work was supported by FAPESP (Fundação de Amparo a Pesquisa do estado de São Paulo), grants number 2016/09452‐0 and 18/02385-0, and FINEP (Financiadora de Estudos e Projetos) grants number FINEP/CT‐INFRA‐01/2011.

## CRediT authorship contribution statement

**Samar Nasser Chehimi:** Formal analysis, Writing – original draft. **Vanessa Tavares Almeida:** Methodology, Validation. **Amom Mendes Nascimento:** Methodology, Validation. **Évelin Aline Zanardo:** Methodology, Validation. **Yanca Gasparini de Oliveira:** Methodology, Validation. **Gleyson Francisco da Silva Carvalho:** Validation, Writing – review & editing. **Beatriz Martins Wolff:** Validation, Writing – review & editing. **Marilia Moreira Montenegro:** Validation, Writing – review & editing. **Nilson Antônio de Assunção:** Funding acquisition, Resources. **Chong Ae Kim:** Resources, Methodology. **Leslie Domenici Kulikowski:** Visualization.

## Conflicts of interest

The authors declare no conflicts of interest.
